# Scrolling to Success: Leveraging Social Media to Highlight Wellness Culture in Orthopedic Surgery Residency Programs

**DOI:** 10.7759/cureus.74846

**Published:** 2024-11-30

**Authors:** Kevin M Posner, Cassandra Bakus, Liem Pham, Geoffrey R O'Malley, Grace Chester, Sophie S Lee, Dante A Implicito, Wayne S Berberian

**Affiliations:** 1 Department of Orthopedic Surgery, Hackensack Meridian School of Medicine, Nutley, USA; 2 Department of Medical Education, Hackensack Meridian School of Medicine, Nutley, USA; 3 Department of Neurosurgery, Hackensack University Medical Center, Hackensack, USA; 4 Department of Orthopedic Surgery, Midwestern University Chicago College of Osteopathic Medicine, Downers Grove, USA; 5 Department of Orthopedic Surgery, Hackensack University Medical Center, Hackensack, USA

**Keywords:** burnout, facebook, instagram, resident education, social media, wellness, x (formerly twitter)

## Abstract

Background

Burnout, characterized by emotional exhaustion, depersonalization, and reduced personal accomplishment, profoundly affects interprofessional collaboration. Despite rising burnout rates, there is a paucity of research regarding the use of social media to support wellness culture, particularly among orthopedic surgery residents.

Methods

A list of all US orthopedic surgery residency programs was compiled through the Accreditation Council for Graduate Medical Education (ACGME) and associated social media accounts were identified. Authors tallied and organized all posts into these categories: work-life balance, attendance to physical health team building activities, healthy work environments, activities or lectures designed to promote wellness, images that imply participating in wellness activities, educational events that include wellness, and resident spotlight. All posts were included up to November 19, 2022.

Results

Out of 197 programs, 110 (55.8%) had a dedicated Instagram account, 10 (5.07%) had a specific Facebook page, and 30 (15.2%) had an X (formerly Twitter) account for their residency programs, generating 13,203 posts for analysis. Across all three social media platforms, posts addressing work-life balance constituted the highest percentage of wellness-related content (n=1211,22.98%), followed by images implying participation in wellness activities (n=1182,22.43%) and promoting a healthy work environment (n=849,16.11%). Specifically, for Instagram, programs ranked within the top 100 on Doximity had significantly more posts and followers compared to those ranked over 100 (92.64 vs. 59.7, p<0.001; 1680.72 vs 1125.02, p<0.001). Additionally, accounts linked to programs with 25 or more residents had significantly more posts and followers compared to those with 20 or fewer residents (97.54 vs. 61.03, p<0.001; 1726.87 vs 1196.32, p<0.001).

Conclusion

Social media possesses substantial potential to promote and emphasize wellness among residents. However, its utilization for this purpose remains underdeveloped, particularly in smaller and lower-ranked orthopedic surgery training programs. Further research is needed to determine the most effective ways to harness online activity to highlight and enhance wellness efforts among trainees.

## Introduction

Burnout is a pervasive psychological syndrome characterized by emotional exhaustion, depersonalization, and diminished personal accomplishment, primarily affecting professionals in roles requiring interpersonal interactions [1]. Medical professionals, particularly residents and fellows, are susceptible to high burnout rates when compared to medical students and the general population. Studies report alarming figures, with 50% of medical students and 51.5% of internal medicine residents experiencing burnout [2-4]. 

Medical burnout has dire consequences, including increased errors, suboptimal patient care, and potential links to physician depersonalization [1,5]. Furthermore, it strains work environments, leading to low morale, absenteeism, and higher job turnover. Personal dysfunction is another facet, with elevated rates of alcohol and substance abuse, insomnia, and relationship turmoil being associated with burnout [5].

The etiology of this problem is complex, with external and internal factors contributing. External factors relate to workplace conditions, including organizational issues, high demands, and poor communication. Internal mechanisms involve self-imposed unrealistic expectations, social isolation, and neglect of personal needs. Burnout is thought to evolve through stages, including a honeymoon phase, the onset of stress, chronic stress, burnout, and habitual burnout, with physical exhaustion typically emerging after stage two and mental/emotional exhaustion following at stage three [6].

Coping strategies, encompassing cognitive and behavioral efforts to manage overwhelming demands, play a critical role in addressing burnout [7]. A multifaceted approach that tackles both external and internal causes is necessary. While more resources can alleviate external stressors, individuals must adopt personal coping strategies, depending on symptom severity. For mild symptoms, interventions promoting work-life balance and discouraging perfectionism may suffice [6]. Weiner et al. identified five categories of preventative strategies among physicians, with an "approach to life" category demonstrating the most substantial improvement in psychological well-being. This category emphasizes positivity, success focus, work-life balance, and related strategies [4]. 

Social media, a modern tool, holds the potential to facilitate these strategies, particularly in the realm of relationships. Social media simplifies connection-building, strengthens family and friend ties, and enables prospective students to engage with faculty. Residency programs can use social media to spotlight self-care activities, featuring faculty engaging in fitness routines, attending non-professional events, and participating in relationship-building exercises, thereby showcasing their commitment to wellness and encouraging participation [4].

It was recently noted in a meta-analysis of 89 independent studies that medical professionals experience higher rates of burnout when compared to the general population, and surgical trainees, specifically, display higher rates of burnout when compared to their non-surgical counterparts [8]. Orthopedic surgery is no stranger to burnout and its cardinal features. Up to 37.2%, 48% and 33.1% of orthopedic residents were found to grapple with high degrees of emotional exhaustion, elevated degrees of depersonalization and diminished sense of personal accomplishment, respectively [9]. Despite the fact that there is a battle being waged on burnout, it is unclear exactly how residency programs, specifically orthopedic surgery residency programs, are working to reduce the rates of burnout. It is known that residency trainees are particularly subjected to the negative impacts that burnout imposes. However, it is still unclear what efforts are implemented by residency programs to reduce the rates of burnout. In response to rising burnout rates, the general public has utilized social media platforms to educate and hypothesize burnout reduction strategies. The medical profession is no exception to this reality. However, there is little data on how social media is leveraged to highlight wellness. Furthermore, there is no data on how wellness can be highlighted among hyper-competitive residency trainees, such as orthopedic surgery. The primary objective of this study is to assess how orthopedic surgery residency programs in the United States utilize social media platforms (Instagram, Facebook, and X) as a tool to highlight and promote wellness among their trainees. Our secondary objective is to analyze the type and frequency of wellness-related content shared on these social media accounts.

## Materials and methods

Program and data collection

To conduct this observational study, a list of all United States orthopedic surgery residency programs was obtained via the Accreditation Council for Graduate Medical Education (ACGME) website.

The ACGME-accredited programs were then cross-referenced with the list of residency programs accepting ERAS applications for the 2022-2023 application cycle in order to arrive at the final study population. The rank and size of each program were then collected using Doximity reputation rankings [10]. 

To obtain each program's accounts, Instagram, Facebook, and X (formerly Twitter) were searched for active accounts. The search was conducted by entering the official name of the residency training program into each social media platform's search bar. Accounts that were found to be solely dedicated to one of the identified orthopedic surgery programs were included. Accounts that were identified but found to be not specifically dedicated to the orthopedic surgery residency program were excluded. These exclusions included accounts related to Orthopedic Department accounts, Department of Surgery accounts, or other surgical subspecialties. Active accounts were defined as accounts that had at least one post during the 2022 calendar year. The authors then reviewed all posted images for each account up until November 18th, 2022. All images posted after November 18th, 2022, were not included. Data was compiled and stored using Microsoft Excel (Microsoft Corporation, Redmond, Washington, USA). 

Content analysis

Posts were analyzed and categorized into seven classes of content based on prior residency initiatives [11-14]. The classifications consisted of posts that displayed (1) work-life balance, (2) attendance to physical health, (3) team building activities, (4) healthy work environments, (5) activities or lectures specifically designed to promote wellness, (6) images that imply but do not directly show residents participating in wellness activities, or (7) educational events that incorporated wellness [11-14]. Two reviewers independently categorized each post based on image content alone. If discordance was found between the reviewers regarding a post, a third reviewer was utilized to make the final categorization. 

For Instagram and X, the number of followers, number of total posts, and number of accounts the page was following were recorded. For Facebook, the total number of posts, the total number of page likes, and the number of accounts the page was following were recorded. 

Descriptive statistics were calculated to summarize the average number of posts across programs and categories. Independent t-tests were used to compare the number of posts and followers between programs based on specific characteristics. Analysis of variance (ANOVA) with Games-Howell post-hoc testing was performed to compare content across social media platforms (Instagram, X, and Facebook), accounting for differences in sample sizes. All statistical tests were performed using SPSS version 25.0 for Mac (IBM Corp., Armonk, New York, USA) with a threshold of significance of P<0.05.

## Results

Of the 197 programs identified, 110 (55.8%) had a residency-specific Instagram, 10 (5.07%) had residency-specific Facebook, and 30 (15.2%) had a residency-specific X (formerly Twitter). Across all three platforms, a total of 13,203 posts were identified and analyzed. Instagram accounted for the most posts with a total of 8,718 (66%), followed by X (formerly Twitter) at 3,869 (29.3%) and Facebook with 616 (4.7%). The average number of posts per program across all platforms was not statistically significant, with Instagram having an average of 79 posts, Facebook with 62, and X (formerly Twitter) with 129 (p=0.066) (Table 1). 

**Table 1:** Social media accounts and total posts used by orthopedic surgery residency.

Platform	Instagram	X (formerly Twitter)	Facebook
Social media accounts	110 (55.8%)	30 (15.2%)	10 (5.07%)
Total posts	8718 (66%)	3869 (29.3%)	616 (4.7%)
Average posts	79	129	62

Of the 8,718 Instagram posts, 4,380 (50.2%) met at least one of the wellness criteria (Table 2). The most common wellness-related posts on Instagram were posts related to work-life balance (n=1,036, 23.7%), followed by images that imply participation in wellness activities (n=1,020, 23.3%), activities or lectures designed to promote wellness (n=693, 15.8%), team building activities (n=570, 13.0%), health work environments (n=565, 12.9%), attendance to physical health (n=404, 9.2%), educational events that included wellness (n=92, 2.1%). 

**Table 2:** Wellness content of posts by orthopedic surgery residency programs.

Wellness criteria	Instagram	X (formerly Twitter)	Facebook
Work-life balance	1036 (23.7%)	154 (20%)	21 (17.8%)
Attendance to physical health	404 (9.2%)	49 (6.4%)	12 (10.2%)
Team building activities	570 (13%)	49 (6.4%)	21 (17.8%)
Healthy work environment	565 (12.9%)	270 (35%)	14 (11.8%)
Activities or lectures designed to promote wellness	693 (15.8%)	48 (6.2%)	21 (17.8%)
Images that imply participating in wellness activities	1020 (23.3%)	135 (17.5%)	27 (22.9%)
Educational events that included wellness	92 (2.1%)	66 (8.5%)	2 (1.7%)
Total posts	4380	771	118

Of the 3,869 X (formerly Twitter) posts, 771 (19.9%) met at least one of the wellness criteria (Table 2). The most common wellness-related posts on X (formerly Twitter) were posts related to healthy work environments (n=270, 35.0%), followed by work-life balance (n=154, 20%), images that imply participating in wellness activities (n=135, 17.5%), educational events that included wellness (n=66, 8.5%), attendance to physical health (n=49, 6.4%), team building activities (n=49, 6.4%), and activities or lectures designed to promote wellness (n=48, 6.2%). 

Of the 616 Facebook posts, 118 (19.1%) met at least one of the wellness criteria (Table 2). The most common wellness-related posts on Facebook were images that imply participation in wellness activities (n=27, 22.9%), followed by work-life balance (n=21, 17.8%), team building activities (n=21, 17.8%), activities or lectures designed to promote wellness (n=21, 17.8%), health work environment (n=14, 11.8%), attendance to physical health (n=12, 10.2%), and educational events that included wellness (n=2, 1.7%).

When analyzing the total number of followers across all three social media platforms, there were significantly more followers on average for each Instagram account when compared to Facebook and X (formerly Twitter), respectively (1,455 vs. 341 vs. 740, p=0.000). 

Table 3 details the mean percentage of wellness-related posts by orthopedic surgery residency programs across Instagram, X (formerly Twitter), and Facebook. Instagram had the highest percentage of posts meeting wellness criteria, such as work-life balance (9.54%) and participation in wellness activities (9.26%). In comparison, Facebook consistently had the lowest percentages. Significant differences were found across platforms for most wellness criteria. 

**Table 3:** Distribution of wellness-related content across social media platforms. P-values for ANOVA tests are listed. P-values for Games-Howell post-hoc testing are not listed. The ANOVA results indicated significant differences; however, no significant differences were found in the post-hoc analysis. ANOVA: analysis of variance.

	Mean percent of wellness-related posts that met the criterion			
Wellness criteria	Instagram	X (formerly Twitter)	Facebook	P-value
Work-life balance	9.54	5.13	2.10	0.004
Attendance to physical health	3.64	1.63	1.20	0.003
Team building activities	5.18	1.63	2.10	0.001
Healthy work environment	5.17	9.00	1.40	0.056
Activities or lectures designed to promote wellness	6.35	1.60	2.10	0.001
Images that imply participating in wellness activities	9.26	4.50	2.70	0.005
Educational events that included wellness	0.84	2.20	0.20	0.002

Resident spotlights

When analyzing each account, the number of posts dedicated to resident spotlights was tallied. There was no significant difference between the average number of resident spotlight posts for each account across the three platforms (Table 4). Resident spotlight posts account for 1,058 (12.13%) of total Instagram posts, 399 (10.31%) of X (formerly Twitter) posts, and 55 (8.92%) of Facebook posts (Table 4).

**Table 4:** Resident spotlight posts.

Platform	Instagram	X (formerly Twitter)	Facebook
Total no. posts	1058	399	55
Percentage of posts (%)	12.13	10.31	8.92
Average no. posts per program	9.7	13.3	5.5

Doximity ranking

**Figure 1:**
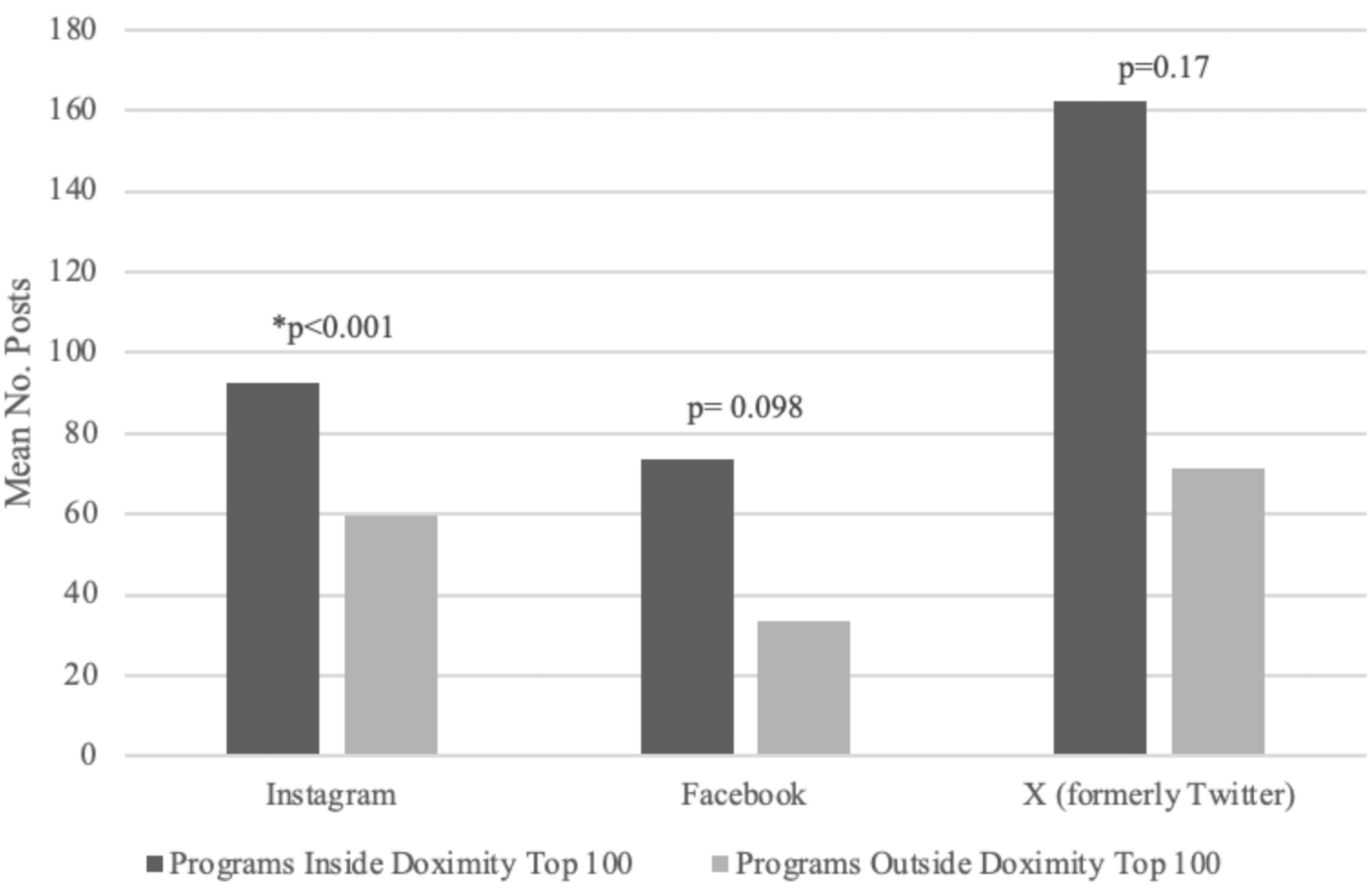
Comparison of average number of posts by program Doximity ranking. *P indicates statistically significant differences between groups.

Accounts were analyzed based on Doximity ranking and split according to those ranked in the top 100, as opposed to those ranked outside the top 100. On average, for Instagram, programs ranked in the top 100 had a higher number of posts when compared to those ranked outside the top 100 (92.64 vs 59.7, p<0.001) (Figure 1). On average, each X (formerly Twitter) account had a larger number of posts per account among those programs ranked in the top one hundred as opposed to outside the top 100 (162.26 vs 71.45, p=0.17) (Figure 1). Facebook accounts, on average, had a higher number of posts among accounts linked to programs in the top one hundred when compared to the programs ranked outside the top 100 (73.57 vs 33.66, p=0.098) (Figure 1).

Residency program size

**Figure 2:**
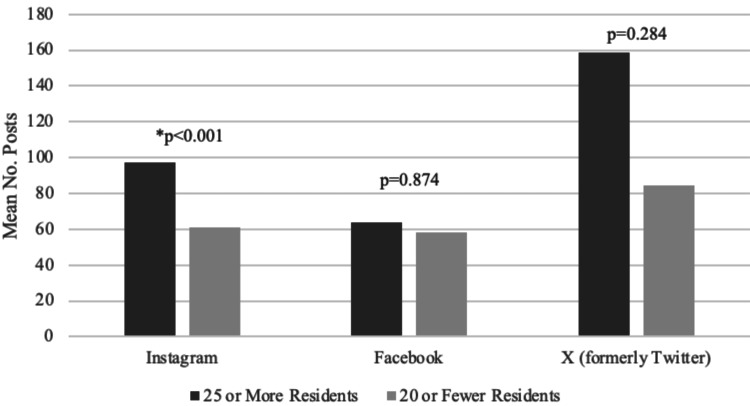
Comparison of average number of posts by residency program size. *P indicates statistically significant differences between groups.

Programs were stratified and analyzed based on the total number of residents (20 residents or fewer vs 25 or more residents total) at each program. On average, Instagram accounts linked to programs with 25 or more residents had a larger number of total posts when compared to residencies with 20 or fewer residents (97.54 vs 61.03, p<0.001) (Figure 2). On average, X (formerly Twitter) accounts linked to programs with 25 or more residents had a larger number of total posts when compared to residencies with 20 or fewer residents (158.77 vs 84.25, p=0.284) (Figure 2). On average, Facebook accounts linked to programs with 25 or more residents had a larger number of total posts when compared to residencies with 20 or fewer residents (64.00 vs 58.00, p=0.874) (Figure 2).

## Discussion

Orthopedic surgery is no stranger to the pervasive phenomenon of burnout, with up to 32.3% of surgeons being affected [15]. Addressing this issue is crucial, as burnout not only impacts the well-being of physicians and trainees but can also have serious implications for patient care, job satisfaction, turnover rates, and absenteeism [1,16].

Physicians are dedicated to providing high-quality healthcare equitably. However, the creeping presence of burnout can hinder this goal, leading to increased medical errors and suboptimal patient care [17-19]. Therefore, it is imperative to identify and treat burnout cases promptly.

Effective prevention strategies encompass both individual-focused and organizational-level interventions or programs [20]. These interventions may include structured small-group sessions, stress management education, and self-care training [20-26]. Furthermore, interventions focusing on enhancing the quality of life outside the hospital, such as mindfulness practices and physical activity, have also shown promise [27,28].

In the context of high-stress residency programs like orthopedic surgery, social media emerges as a valuable platform for promoting wellness activities. Medical students applying for orthopedic residencies prioritize factors like the perceived happiness and quality of life of current residents, camaraderie among residents, and impressions after away rotations [29]. This study provides a thorough examination of wellness-related content as it analyzes posts across multiple social media platforms from 197 orthopedic residency training programs, offering a broad understanding of the information displayed. In recognizing the importance of wellness in orthopedic programs, it's noteworthy that social media accounts predominantly share wellness-related content on Instagram (50.2% of posts). In contrast, according to our criteria, X and Facebook appear less active in posting wellness-related content. Recent data indicate that Instagram is the most common social media application used by orthopedic residency applicants, with 69.7% of respondents using it to learn more about programs [30]. Surprisingly, only 110 (55.8%) of orthopedic residency programs have a residency-specific Instagram account. This underutilization of social media represents an opportunity for orthopedic training programs to not only advertise their residencies but also to convey the wellness-related activities they offer. Fostering a culture of wellness and implementing wellness initiatives within orthopedic surgery residency programs is essential to prevent burnout. It is worth noting that plastic surgery, neurosurgery, and anesthesia residency programs have demonstrated significant efforts to reduce burnout through integrated wellness programs, utilizing social media as one of their tools in this battle.

The inclusion of an evaluation of programs based on rank and size adds an additional dimension to the observed findings, providing further depth to the information compiled. When evaluating programs based on Doximity rankings and program size, it becomes evident that higher-ranked and larger programs tend to use social media more extensively, with Instagram being particularly prominent. This raises the question of whether smaller and lower-ranked programs have wellness initiatives or foster a culture of wellness among their residents. While our study does not provide conclusive evidence on this matter, it highlights that larger and higher-ranked programs have a broader reach in influencing applicants and the medical community. The existence and effectiveness of wellness programs in smaller residencies remain uncertain, as certain factors that contribute to wellness culture may not be adequately represented on social media.

A closer look at program accounts reveals various wellness initiatives. These include efforts to highlight diversity among programs, outdoor physical activities like hiking, biking, and swimming, promoting work-life balance through attendance at sporting events or dining with peers, and celebrating both small and significant achievements of residents. Team building activities, such as first-year interns collaborating to master casting techniques or attendings and students engaging in research discussions over meals, are also commonly showcased.

While social media has undoubtedly revolutionized communication, it is not without drawbacks. Although a direct causal link has not been established, social media usage is believed to contribute to the rise of mental health disorders such as anxiety and depression [31]. The impact of social media on burnout remains a complex topic. Some studies have identified a positive correlation between social media use and burnout levels. Excessive social media use can adversely affect emotional well-being, relationships, physical health, and overall performance [32]. Users who engage in social comparison often report higher rates of emotional exhaustion, reduced sense of accomplishment, and burnout [33,34]. However, it is crucial to acknowledge that individuals who experience negative symptoms often use social media inappropriately or excessively. When used appropriately, social media can alleviate work-related stress, reduce emotional exhaustion, and combat feelings of alienation by fostering social connections [11,35,36]. Therefore, judicious and responsible social media use can offer benefits to users.

Nevertheless, it is essential to exercise caution when sharing information online. Through this discussion, we hope to encourage orthopedic residency programs not to misrepresent their wellness initiatives on social media. While social media cannot provide a perfect representation of the culture within an orthopedic residency program, given its global reach, we believe that programs should consider creating dedicated social media accounts to promote the importance of wellness in medicine. Such efforts can contribute to creating healthier workplaces for orthopedic surgery residents and future surgeons.

In the future, programs should continue to leverage social media as an essential tool for highlighting wellness culture and connecting with residents. However, their efforts should extend beyond social media alone. Programs must persistently combat the pervasive culture of burnout in medicine and orthopedics by organizing wellness-driven events and implementing comprehensive wellness programs. It is crucial to strike a balance, ensuring that these initiatives do not inadvertently harm residents or younger medical trainees attempting to utilize social media to their advantage.

Limitations

There are a number of limitations to this study. The largest limitation is the cross-sectional nature of this study. There are a large number of different social media platforms, all of which are ever-changing and fluid environments with followers, likes, views, and posts constantly updating in real time. This factor was mitigated by limiting the time frame in which posts and accounts were analyzed. Despite this, not all social media platforms available to the public were queried, possibly resulting in missed posts/content related to wellness. It is also worth noting that the posts or content on social media accounts do not necessarily reflect the true amount of engagement in wellness activities. Therefore, while social media offers insights into how programs highlight wellness initiatives, it may not fully capture the extent to which these initiatives impact residents. We were also limited by the fact that many residency programs have accounts that are combined with general orthopedic departments or general surgery departments, limiting the total number of posts that were able to be analyzed. With regards to the posts themselves, while categorization was conducted in a systematic fashion to reduce subjectivity, the categories utilized may inherently be interpreted in different ways. This was mitigated to the fullest extent possible by utilizing two independent reviewers, with a third reviewer acting as the final decision-maker in times of discordance. Finally, we were limited by the geographic scope of our search. Only orthopedic training programs in the United States were included in this study. Therefore, the findings may not be generalizable to orthopedic training programs in other countries, where cultural differences and varying approaches to wellness and social media utilization could influence the results.

## Conclusions

It is clear that social media may be wielded as a powerful tool to highlight wellness among residents. Specifically, Instagram proved to be a widely utilized platform with the most posts and followers when compared to Facebook and X. Despite this, the usage of social media for this specific purpose in orthopedic surgery training programs is not being exploited to its fullest extent. Further investigation is needed to clarify whether a causal relationship exists between social media content and resident wellness, as the current findings show only a correlation and do not directly assess whether residents engage with or derive benefit from the content, despite disparities in social media utilization between larger, higher-ranked programs and smaller, lower-ranked ones. This will allow programs to determine the most effective way to leverage online activity to promote wellness among residents. The high prevalence of burnout among orthopedic surgeons and residency trainees makes it imperative that physician wellness is cultivated and promoted in the medical community.
